# The Role of Tumor Suppressor p53 Protein in HIV–Host Cell Interactions

**DOI:** 10.3390/cells13181512

**Published:** 2024-09-10

**Authors:** Mary Bakhanashvili

**Affiliations:** The Mina and Everard Goodman Faculty of Life Sciences, Bar-Ilan University, Ramat-Gan 5290002, Israel; bakhanus@yahoo.com

**Keywords:** HIV, p53, DNA repair, proofreading

## Abstract

The virus–host relationship is indispensable for executing successful viral infection. The pathogenesis of HIV is determined by an intricate interaction between the host and the virus for the regulation of HIV infection, thereby influencing various aspects, including the regulation of signaling pathways. High mutation rates and population heterogeneity characterize HIV with consequences for viral pathogenesis and the potential to escape the immune system and anti-viral inhibitors used in therapy. The origin of the high mutation rates exhibited by HIV may be attributed to a limited template-copied fidelity that likely operates in the cytoplasm. HIV-1 infection induces upregulation and activation of tumor suppressor p53 protein in the early stages of HIV-1 infection. p53 plays a multifaceted role in the context of HIV infection, thereby affecting viral replication. p53 is involved in maintaining genetic integrity, actively participating in various DNA repair processes through its various biochemical activities and via its ability to interact with components of the repair machinery. This report focuses on the impact of the p53 protein on the HIV-1 reverse transcription process while incorporating various incorrect and non-canonical nucleotides. The presence of functional host-coded p53 protein with proofreading–repair activities in the cytoplasm may lead to various biological outcomes.

## 1. Introduction

The human immunodeficiency virus type 1 (HIV-1) is a lentivirus (a subgroup of retrovirus) that causes a progressive loss in numbers and functions of CD4+T lymphocytes, the regulators of the adaptive immune system [1]. Apoptosis is one of the presumptive causes of CD4+T cell depletion and progression to acquired immunodeficiency syndrome (AIDS) [2]. HIV-1 is unique among the retrovirus family, as the virus can infect and replicate in two target cells: dividing cells (e.g., activated CD4+T) and non-dividing cells (e.g., terminally differentiated macrophages) [3,4]. While persistent HIV-1 infection invariably causes progressive depletion of CD4+ T cells, macrophages, HIV-infected long-lived cells, in contrast to lymphocytes, are less-prone to virus-mediated cytopathic effect-apoptosis, thereby supporting sustained persistent virus expression in an infected individual, increasing the time frame for the release of virus particles from these cells [5]. Moreover, human macrophages, serving as a durable cell reservoir of HIV-1, contribute to the lower susceptibility to antiviral therapy, creating a barrier to viral eradication [6,7,8]. Virulence, pathogenesis, and the capacity to develop active anti-retroviral drugs and vaccines mainly depend on genetic diversity observed in viruses, both between patients and within each individual [9,10]. The differential susceptibility of cells to HIV-1 infection may be affected by host factors. The ensuing host–virus interactions mediate the activation of cell intrinsic factors and define the outcomes of the responses, e.g., the death of the target cell. During the viral infection, many cellular proteins change their sub-cellular localization with the subsequent expression of pro-viral role or anti-viral activities. Identification of host factors and their roles during infection is crucial for understanding cell–virus communications, which might provide new therapeutic targets for efficiently controlling viral replication in these cells and limiting the formation of reservoirs in exposed individuals. Notably, HIV-1 exhibits exceptionally high mutation frequencies. Mutations produced during proviral DNA synthesis is the most pertinent basis of the emergence of virus sequence variation [11,12]. Interestingly, tumor suppressor p53 protein displays multiple activities in DNA repair [13]. The primary objective of this review is to clarify the role played by cellular p53 protein during the course of HIV infection, with a particular emphasis on its capacity to affect the accuracy of proviral DNA synthesis in the cytoplasm. Information on HIV and target cell interplay is essential for the creation of a comprehensive scene of virus replication within the host cellular milieu and may open up the avenues of establishing novel anti-HIV drugs in the future.

## 2. HIV Replication

**Figure 1:**
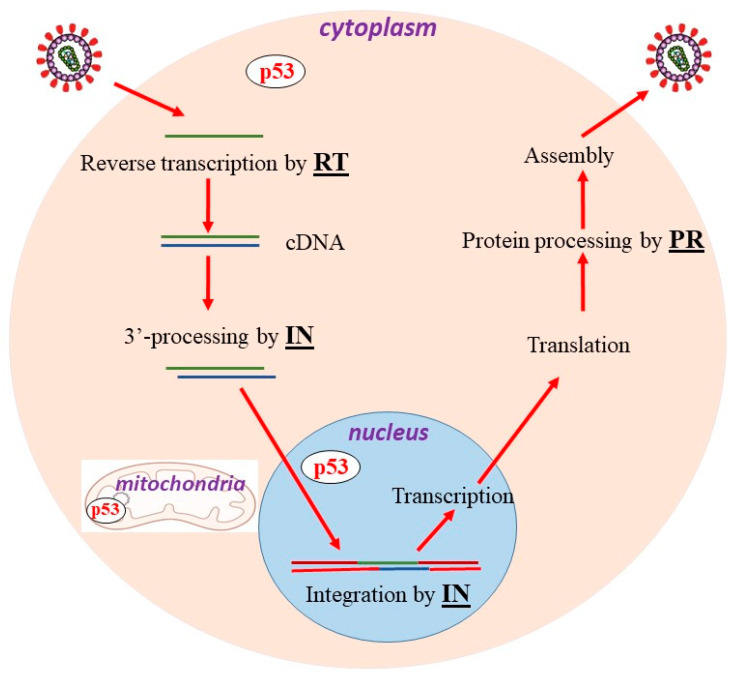
HIV life cycle. The participation of viral encoded proteins during the unique steps of HIV replication: reverse transcription by reverse transcriptase (RT) in cytoplasm, 3′-processing of proviral DNA by integrase (IN) in cytoplasm, integration of proviral DNA into host genome by IN in nucleus, viral protein processing by viral protease (PR). p53 is localized in cytoplasm, mitochondria, and nucleus. See the manuscript text for details.

All viruses rely on and manipulate the molecular mechanism of their host’s cells for successful replication [14]. The pathogenesis of HIV is determined by an intricate interaction between the host and the virus for productive viral infection and initiation of the viral life cycle [15]. The HIV genome comprises two identical positive-strand RNA containing two regulatory genes (tat and rev), three structural genes (gag, pol, and env), and four accessory genes (nef, vpr, vpu, and vif) [11]. Retroviruses are RNA viruses that multiply through a DNA intermediate, exclusive to the retroviral life cycle [11]. The HIV life cycle consists of various steps: binding, attachment, fusion, trafficking, reverse transcription, nuclear import, integration, transcription, translation, assembly, budding, maturation, and release (Figure 1). HIV-1 infection is broadly categorized into early and late replication stages, accompanied by its own “viral enzymes” (encoded by the *pol* gene), such as reverse transcriptase (RT), integrase (IN), and mature protease (PR), which catalyze key phases of the viral replication cycle.

(a) Following HIV entry into target cells, such as macrophages and CD4+ T cells, and capsid disassembly, the viral-coded enzyme RT converts the genomic material–viral ssRNA into proviral dsDNA in the host cytoplasm, which is then ultimately integrated into the host genome in the nucleus [16]. The provirus serves as the genetic element for the production of progeny virions during the late steps of infection.

(b) Integrase facilitates the integration of the reverse-transcribed proviral DNA into the infected target cell DNA, which is an essential and irreversible event in the replication cycle and the pathogenesis of HIV [17]. The integrated DNA becomes the genetic content that provides the template for further viral protein production. Cells, with integrated proviruses, may persist for many years, undergo clonal expansion, and produce replication competent HIV. Even proviruses with defective genomes can yield HIV RNA and may contribute to ongoing HIV pathogenesis [17]. Viral integration causes damage to the target cell’s DNA and triggers cell killing. The integration proceeds by two consecutive steps: (*i*) removal of two nucleotides from the 3′-ends of the viral cDNA after reverse transcription in the cytoplasm and (*ii*) integration, namely, strand transfer of the viral DNA template into the target host’s DNA in the nucleus [18] (Figure 1). Notably, the 3′-prime processing makes the viral DNA vulnerable to auto-integration in the cytoplasm, a suicidal side pathway known to be catalyzed by IN, as well [17]. Auto-integration is a problem faced by retroviruses, as self-integrating end-products could be generated. 

(c) Subsequent HIV replication steps such as gene transcription, nuclear export of viral RNAs, and mRNA translation into the polyprotein relies on host machinery for gene expression (Figure 1). Viral polyprotein would then be cleaved into individual functional proteins by the HIV-1 protease to create the mature protein components of an infectious form of HIV viral particles (including a newly synthesized HIV-1 PR). All these steps represent key late events of the replication cycle in the cytoplasm [19]. The viral proteins, along with the newly synthesized viral RNA, assemble into progeny virions, which bud off the target cell and mature into newly infectious viral particles.

During the successful HIV-1 life cycle, viral components cooperate with various cellular proteins and exploit them for multiple purposes throughout their intracellular replication [15]. HIV-1 replicates by utilizing numerous cellular processes via up-regulation or down-regulation of specific host proteins that control viral pathogenesis [20]. Consequently, the cellular proteins can either suppress or assist HIV-1 replication, thereby serving as negative (or restriction) or positive co-factors of viral replication [21]. Viral infections, including HIV, are stressful, and apoptosis can be elicited by the incoming viral genomic RNA in host cells. Macrophages, in contrast to lymphocytes, are intrinsically resistant to HIV-induced cytopathic effects and less susceptible to some anti-retroviral drugs [5,8]. The differential susceptibility of target cells to HIV-1 infection may be affected by host factors, and the tumor suppressor p53 protein has been found to play an important role. Notably, p53 displays opposite effects in accordance with cell type by triggering apoptosis or by regulating the expression of definite target genes, allowing the cells to survive [20]. Promoting apoptosis in response to various stress signals is reflected through the “canonical” transcriptional function of p53, which is dominant in tumor suppressor activity [21]. 

High mutation rates, population heterogeneity, and error-prone reverse transcription are characteristic features of HIV infection with the subsequent consequences associated with viral pathogenesis and the potential to escape the immune system and anti-viral inhibitors utilized in therapy [13]. Genetically heterogeneous virus populations arise constantly, varying in relative frequency as viral replication continues, which is essential for virus adaptation to changing environments. The high viral mutation incidences of viruses impede the prevention and therapy of AIDS [15,22]. The source of the high mutation rates revealed by HIV may be attributed to an inherent low polymerization accuracy by HIV-1 RT during the process of reverse transcription that operates during the early steps of HIV replication in the cytoplasm (Figure 1) [12,23,24,25]. HIV-1 RT is a multi-functional enzyme displaying the three RT-associated activities that catalyze the necessary steps of proviral DNA synthesis [12]: (a) transcription of viral RNA into the full-length minus-strand DNA by the RNA-dependent DNA polymerization function; (b) the hydrolysis of RNA from RNA-DNA heteroduplexes by the RNase H activity, and (c) copying of the minus-strand DNA into the new second plus-strand DNA by the DNA-dependent DNA polymerization function. The high error rates are created during both minus-strand (with an RNA template) and plus-strand (with a DNA template) DNA synthesis due to the efficient misinsertion of non-complementary nucleotides and higher primer-mismatch extension proficiency after a misinsertion event by HIV-RT [23,24,25,26]. In addition, the absence of intrinsic proofreading–repair activities (e.g., 3′→5’ exonuclease function), as part of HIV-1 RT to correct the errors introduced into the proviral DNA, contributes to mutagenesis [23,24]. Possibly, both the efficient dNTP-binding affinity and proficient DNA-synthesis skill of HIV-1 RT are likely to underpin its error-prone polymerization, thus supporting the high mutation rate of HIV-1 and the continues production of new HIV variants. Notwithstanding, the limitation of error rates is anticipated to preserve the encoded genetic information and avoid fitness losses of viruses bearing genomes with multiple mutations. 

Within the replication complex, cells can repair a large variety of DNA lesions through a diversity of sophisticated DNA repair pathways, recognizing and activating a battery of proteins/factors for error-editing functions, e.g., tumor suppressor p53 protein [15]. Remarkably, HIV infection evokes cellular stress, and the infected cells harbor the high levels of activated p53 protein possessing various DNA repair activities. Indeed, p53 in cytoplasm was found to influence the accuracy of DNA synthesis by retroviral RTs, raising the possibility for the role of p53 in maintaining retroviral genomic integrity [27].

## 3. Characterization of Tumor Suppressor p53 Protein

The tumor suppressor p53 protein plays a pivotal role in controlling various aspects of health and disease [28,29]. The p53 protein is an essential regulator of various cell signaling pathways, displaying functional heterogeneity in its non-induced state and under various p53 inducible conditions. Under normal conditions, p53 expression levels are low, but they are elevated under a broad range of stress conditions. A low p53 level is maintained by murine double minute 2 (MDM2), an E3 ubiquitin ligase, targeting p53 for proteasomal degradation [30,31]. However, the accumulation and functional activation of p53 occurs in response to a multitude of intra- or extra-cellular stressful stimuli (e.g., DNA damage, replication stress) [32]. The induction of p53 is predominantly accomplished by disconnecting the p53–mdm2 association [31]. Markedly, p53 in the cytoplasm, through the expression of mdm2-independent inherent “RNA decay” function, might act as a negative regulator of p53-mRNA levels, thereby affecting the cellular levels of p53, which could potentiate or attenuate the p53 biological response to stress signals [33,34].

**Figure 2:**
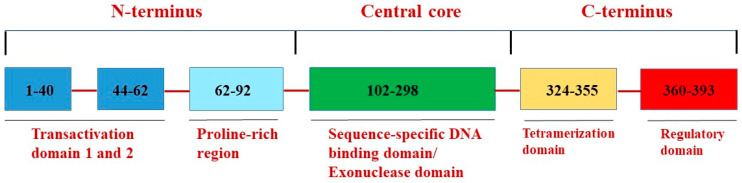
Schematics of functional domains of p53. Functional domains are indicated below the diagram.

The p53 protein consists of 393 amino acids and five main functional regions (Figure 2): (a) the transactivation domain, subdivided into two regions: TAD1 and TAD2, is located at the N-terminus, allowing the binding of p53 to different co-factors; (b) proline-rich domain, which is dispensable for the ability of p53 to bind to DNA; (c) the central core region residues 102 to 292 contains two independent biochemical activities, i.e., sequence-specific DNA binding and non-sequence-specific 3′→5′ exonuclease activity; (d) tetramerization domain, located at the C-terminus, allows four p53 proteins to oligomerize as a tetramer, permitting the appropriate protein conformation to bind to DNA for sequence recognition; (e) the regulatory domain transiently lets p53 bind to non-specific DNA sequences [35]. 

The ensuing increase in p53 level provokes numerous biological responses, ultimately leading to transient or permanent growth arrest, apoptotic cell death, and DNA repair [32]. p53 participates in various cellular processes comprising cell cycle arrest at the G1/S and G2/M checkpoints (if the damage is sustainable, allowing time for the cells to repair DNA), or apoptosis (for eliminating cells that contain excessive and irreparable damaged DNA). These processes together defend the organism from the production of genetically unstable cells. The irrefutable role of p53 function appears to participate in protection of the DNA integrity through its “canonical” function, acting as a transcriptional activator of the genes involved in specific cell responses according to the stress type, thereby controlling the cell’s fate [29,36]. Notably, activated p53 may also activate apoptosis through transcription-independent mechanism via the interaction of p53 with anti-apoptotic proteins located in the mitochondria [37].

Interestingly, p53 may also participate directly in DNA repair by its biochemical inherent “non-canonical” 3′→5′ exonuclease function unrelated to transcriptional activity [38,39,40,41]. p53 exonuclease and sequence-specific DNA binding, required for its transactivation function, are separate activities of the p53 core domain, regulated in the opposing manner (Figure 2). Under normal conditions, p53 participates actively in repair processes through activities un-related to sequence-specific DNA binding, specifically through its exonuclease activity. Stress-mediated stimulation of p53 function leads to the activation of sequence-specific DNA binding and transcription-dependent mechanisms, which in turn correlates with a decrease of p53 exonuclease activity [42].

The p53 protein is situated in various sub-cellular compartments, dependent on the cellular stress milieu: the nucleus, cytoplasm, and mitochondria [27,28,37], decoding different signals a cell generates in response to various stress situations. The multi-compartmental effects of p53 are determined by the context-dependent actions of p53 according to the abundance of p53, the type and severity of DNA damage, the nature and intensity of stress signals, and its subcellular localization (nuclear/cytosolic/mitochondria), with multiple post-translational modifications affecting its shuttling between the sub-cellular compartments and its interaction with other cellular or viral proteins [43,44,45,46]. Shuttling between the cellular compartments not only regulates protein localization but also often impacts on protein function in important biological processes.

During cell–virus interactions at the molecular level, target cells utilize intrinsic proteins to identify viral infection and eliminate further viral spreading. Conversely, viruses have developed diverse strategies to subvert host mechanisms that impede viral replication. Strikingly, in addition to being a tumor suppressor protein, p53 may be considered as a virus suppressor protein, as various viruses are known to counter p53 function, employing anti-p53 strategy, either by its sequestration or through degradation using cellular ubiquitination machinery [47]. Increased p53 levels have been noted following the infection of cells with different viruses: SV-40 [48], adenovirus [49], Epstein–Barr virus [50], and HIV [51]. 

Numerous studies have reported the significance of p53 in the process of HIV-1 infection [21,52]. p53 plays a multifaceted role throughout the HIV infection that initiates its activation until the termination of the stress response, interacting with various facets of the HIV life cycle, thus affecting viral replication and disease progression. HIV-1 infection promotes the expression of p53, inducing upregulation and activation of p53 in the early stages of infection in lymphocytes [5]. p53 has been found to conquer HIV infection through various mechanisms: (a) p53 inhibits HIV-1 long terminal repeat (LTR) promoter activity and suppresses transcription from the integrated HIV-1 proviral genome [52]; (b) p53 suppresses Tat protein, a major transactivator of HIV-1 [53]; (c) p53 activates apoptosis of HIV-1-infected immune cells [54]; and (d) p53 and its downstream gene p21 play a significant role in the restriction of HIV-1 early stage replication in target cells [55]. 

HIV-1, through its viral proteins, can inhibit or stabilize various host protein factors to favor a productive infection. Numerous HIV-1 proteins have been reported to interact with p53, with diverse consequences [56]. HIV-1 nef either reduces the p53 expression or suppresses the function of p53 by binding to transactivation domain of p53 in the N-terminal region, thus impeding its DNA-binding activity and transcriptional potential with subsequent inhibition of the p53 apoptotic function [57]. Conversely, HIV-1 env, vif, and vpr can activate the p53 pathway to induce the apoptosis of target cells [56]. Studies on HIV-1-infected primary macrophages have shown that HIV-1 env regulates apoptosis by inducing p53 phosphorylation. Notably, p53 is stabilized and activated by vif. This effect induces G2 phase arrest in infected cells, thereby supporting HIV-1 replication, which demonstrates the negative impacts that p53 has as an antiviral factor against HIV replication.

Remarkably, HIV with the ability to decrease host p53 expression would diminish cellular amounts of type 1 interferons (IFNs) and thereby escape its antiviral effects [58]. p53 is considered a regulator of both innate and adaptive immunity, directly transactivating many basic downstream genes important for immune signaling pathways, producing IFNs, e.g., interferon stimulated gene 15, (ISG15) and protein kinase R (PKR) [58]. Some ISGs are able to recognize viral RNA replicative intermediates and impede viral replication by hydrolyzing viral RNA or inhibiting its translation to decline the viral proteins amounts in the target cell. It was suggested that p53, as part of cellular defenses, in addition to being a “guardian of the genome”, may also serve as a “guardian of immune integrity” [59]. 

Of note, the activation of p53 by HIV infection is triggered by two main DNA damaging events in cells: the reverse transcription process in the cytoplasm and provirus integration into genomic DNA in the nucleus [12,60]. Accordingly, these two processes are to be responded to and responses programed properly, and in this context the sub-cellular location of p53 during the infection is relevant to the p53-mediated response. Since viral infection induces cellular stress, the activated p53 may encourage a biochemical program promoting proviral DNA repair.

## 4. The Impact of p53 on HIV Reverse Transcription Process

The chief biological routes regulated by p53 comprise the preservation of the genomic integrity of cells [61]. The p53 protein exerts various biochemical activities directed at maintaining the genomic stability and accuracy of DNA synthesis by repairing damaged DNA [42]. To protect the integrity of their genetic material, cells are assisted by intricate networks of protein–DNA interactions. Various biochemical activities of p53 related to direct involvement in DNA repair are associated with transactivation-independent functions via non-sequence specific binding to DNA, including nucleotide excision repair (NER) [62] and base excision repair (BER) [63] and 3′→5′ exonuclease activity [42]. 

The excision of erroneous nucleotides from the nascent DNA is a process called proofreading [64,65]. In various cell compartments, augmented DNA replication accuracy is a fundamental feature for conserving genomic integrity. Proofreading can operate in two different ways: ‘intrinsic’ proofreading by DNA polymerase associated 3′→5′ exonuclease activity permits mispair exclusion without intervening enzyme dissociation [64]. Alternatively, the errors can be corrected by ‘extrinsic’ proofreading contributed by other DNA polymerase or damage-repair proteins, comprising the dissociation of the mismatch from the polymerase active site followed by transfer to the exonuclease active site of other proteins [64]. 

In the context of DNA repair, p53, as a cellular factor, plays an important role in successful genome editing. The recombinant and endogenous p53, in various compartments of the p53-proficient cell lines, possesses an intrinsic 3′→5′ exonuclease activity that can be connected to its biochemical function in safeguarding genome stability [62,63,64,65]. By providing an external exonuclease activity, p53 facilitates the correction of mistakes produced by either cellular exonuclease-deficient (e.g., DNA polymerase α in nucleus) [41] or -proficient (e.g., DNA polymerase γ in mitochondria) [66,67] and viral DNA polymerases (e.g., murine leukemia virus RT, HIV-1 RT in cytoplasm), thus operating as a fidelity factor in various compartments of cells [45,46]. 

### 4.1. Removal of Non-Complementary Nucleotides by p53 Protein

Mutations in the HIV genome are a phenomenon generated by errors triggered by the existence of unbalanced deoxyribonucleotide triphosphate (dNTP) concentrations, non-canonical nucleotides, e.g., ribonucleotides (rNs) and uracils (dU), and by environmental factors that threaten the stability of the genetic information [46,68,69]. Cellular responses to various types of DNA lesions include repair processes that act coordinately before, during, and after DNA replication to conserve genomic stability. The polymerization errors (e.g., mispairs, rNs, dU), generated by HIV-1 RT during proviral DNA synthesis in cytoplasm, all have the potential to exhibit DNA damage signals that motivate the host DNA repair pathway [23,68,69]. Following the incorporation of non-complementary nucleotide, the delayed replication course by HIV-1 RT would provide sufficient time for the exonuclease to efficiently erase the 3′-terminal wrong nucleotide [46]. Proviral DNA synthesis in the cytoplasm, as a particular stress signal, initiates a DNA repair path, facilitated by specific protein–DNA interactions, resulting in improved fidelity. p53, by DNA damage recognition capacity, resolves the damage fulfilling functions obligatory for DNA repair, i.e., remove the erroneous nucleotides produced by HIV-1 RT within cytoplasm at the sites of HIV replication [46]. The impact of p53 as a fidelity factor is two-fold: to erase preexisting 3′-terminal mismatches and to preclude the elongation of 3′-mismatched primer ends by the polymerase [40]. Indeed, p53 was found to support an efficient proofreading of base–base mismatches produced during DNA synthesis, which impede the incidence of the mistakes by excising imperfectly inserted mispairs from both RNA/DNA and DNA/DNA substrates in the direct exonuclease assay when first binding to a mispaired 3′-terminus independent of HIV-1 RT and during ongoing DNA synthesis in vitro with both template-primers [40,45]. The participation of p53 in error correction may be based on its direct interaction with DNA (as a general 3′-terminal mismatched DNA binding protein) [70] and biochemical (an inherent 3′→5′ exonuclease) function [40]. The physical association of HIV-1 RT and p53 proteins in the reverse transcription complex may occur via DNA–protein interactions and not via protein–protein (HIV-1 RT/ p53) or DNA–protein–protein interactions [70]. Gel retardation experiments revealed that there was no evidence for the production of a super-shift complex of p53/RT/DNA [70]. Successful repair of DNA lesions depends on the synergistic action of the DNA polymerization system, namely, effective and sequential action of HIV-1 RT and proofreader-competent p53 component of the DNA repair path. According to the suggested framework for understanding the mechanisms of a potential functional collaboration of p53 and HIV-1 RT, the un-extended 3′-terminal mispaired DNA, created following misincorporation, may be recognized by p53 [70]. After the exclusion of the wrong nucleotide, the corrected primer could be transferred to the RT and undergo a rebinding process by the HIV-1 RT, with a subsequent DNA elongation [70]. Coordinating DNA repair by p53 during the DNA synthesis by HIV-1 RT ensures that the DNA is copied with minimum errors. Notably, the same relative order of substrate preferences for mispair removal obtained during DNA synthesis by recombinant HIV-1 RT in cytoplasmic lysates of p53 harboring cells, and in reconstituted reactions, validates the reliability of these observations, thus signifying that the specificity mirrors the proofreading potential of replication apparatus.

Fascinatingly, in cell-free studies the error rates of purified HIV-1 RT are approximately 15–20-fold higher than the mutation rate of HIV-1 in infected cells with the same target sequence [71]. Apparently, the viral or host protein can influence the reverse transcription process in the cytoplasm of infected cells. The HIV-1 regulatory vpr protein has been proven as a viral protein that affects the in vivo mutation rate, since mutation of the vpr gene resulted in a 5-fold decrease in HIV-1 mutation rate compared to the error rates observed in vitro [72]. Excitingly, purified HIV-1 RT in the presence of p53-harboring cytoplasmic extracts revealed a decrease of up to 15-fold in the mispair extension efficiencies, which coincides with a similar reduction in HIV-1 mutation rate perceived in vivo [46]. p53, executing its extrinsic proofreading feature in the cytoplasm, provides the host-derived DNA repair mechanism during a misinsertion event produced by HIV-1 RT [46]. 

Among the base substitution mutations in the HIV genome, 80% are transitions and 20% are transversions [22]. The functional communication of the fidelity-enhancing cellular constituent p53 with the error-prone HIV-1 RT holds the potential to affect the base substitution specificity. The accuracy of DNA synthesis mirrors multi-faceted collaborations between the parameters of the catalytic “triad” involved in DNA synthesis: DNA polymerase, the nature of the mispair, and fidelity–enhancing accessory components [73]. Understanding the specificity of the mispair excision by p53 is to appreciate the differences in the functional interplays between the viral DNA synthesis (by HIV-1 RT) and DNA damage repair (by p53) with distinct mispaired substrates and their importance to the final outcome. Apparently, the trend in the extension and excision spectrum created are different for polymerization by HIV-1 RT and exonucleolytic degradation by p53 reactions, since the favored production and extension of transition mispairs over transversion mispairs was perceived by HIV-1 RT [64]. By preferential correction of some misincorporated bases to reduce the rates of transversions, p53 may impact the mutation spectra of HIV-1 RT by serving as an external proofreader [46]. 

The mutational spectra and mutation frequencies during DNA synthesis of HIV genetic material probably depend on the precise sequence content and cellular context during the coping template and location of mispair, as each position provides a new set of protein–DNA contacts and neighboring nucleotide sequences may influence recognition of the altered geometry of the mismatch by the enzyme/protein responsible for the proofreading efficacy [74]. It has been proposed that the high A-T content of the primer-terminus compared with high G-C content raises excision rates by assisting the strand separation process [27]. Essentially, the specificity of the mutations observed across the whole viral genes copied may be affected by p53 extrinsic proofreading.

It is imperative to comprehend how to relate the biochemical properties of p53 with its biological behaviors in cells. The accuracy of DNA synthesis by the HIV-1 RT may respond to alterations in the composition of replication complex in the cytoplasm. Different functional subclasses of p53 can exist within the same cell. It is highly expected that p53 function may be guided by sub-cellular localization of the protein. Plausibly, an attractive likelihood is that, in HIV-infected cells, reverse transcription process may facilitate a moderate accumulation of p53 in cytoplasm with preferential expression of exonuclease activity through the non-sequence-specific DNA binding capacity of the protein [46,70]. The rise of the error-correction functions occurs through transient collaboration of p53 with viral DNA replication complex in response to DNA lesions that arise during the dynamic reverse transcription process in the cytoplasm with p53 component binding and dissociating the DNA polymerization complex, thus affecting the polymerase (RT)/exonuclease (p53) ratio [46]. However, the integration of proviral DNA into cellular DNA in the nucleus may induce the accumulation of high levels of p53 activated for sequence-specific DNA binding capability, leading to the preferential triggering of specific target genes involved in apoptosis, influencing the process of reverse transcription.

### 4.2. Excision of Non-Canonical Nucleotides by p53 Protein

In addition to inserting non-complementary nucleotides during genomic DNA replication, another form of replication lesions arise, incorporating non-canonical nucleotides carrying the correct base, but the wrong sugar at substantial rates [75]. The contamination of rNs in DNA can be either integral or non-integral. Integral rNs are produced by direct misincorporation into nascent DNA by DNA polymerases that are unsuccessful in discriminating rNTPs from dNTPs under conditions of a high rNTP/dNTP ratio in cells. Non-integral rN contamination arises following the base-pairing of non-translated portions of RNA transcript with template DNA, thus constructing a replacement loop (R-loop) [75]. The mutagenic incorporation of rNTP is one of the most common threats to genomic stability. The stalling of the HIV-1 RT due to the efficient insertion of rNs, combined with the deficiency of inherent error-correction activities, provides the signal for proofreading by an extrinsic exonuclease [76]. p53, as a fidelity factor, is capable of serving as a driver-proofreader and participating in a 3′-terminal ribonucleotide excision repair pathway. During RNA-triggered DNA damage, p53, by possessing the compatible biochemical properties, is pertinent for editing mistakes created by HIV-1 RT during the diverse steps of rN insertion events through intrinsic pathways (i) by the elimination of pre-existing 3-terminal rN, (ii) by precluding elongation of a 3′ rN-terminated primer during ongoing DNA synthesis, and (iii) by diminishing the stable incorporation of rNs [77]. A positive connection has been perceived between the existence of cytoplasmic p53 and a decline in the stable insertion of rNs in nascent DNA with p53-harboring lysates, e.g., HCT116 cells [77]. The fact that p53 in the cytoplasm can edit an incorrect sugar, irrespective of the identity of the base, magnifies the impact of the p53-proofreader in the repair of DNA lesions by the excision of both a base–base mispair and an improper sugar. Notwithstanding, p53 has the remarkable inherent functionally specialized capacity to recognize both correctly and incorrectly paired rNMPs embedded in DNA, thus letting p53 excellently diminish the mutagenic potential of rNs errantly incorporated into DNA [77]. p53 exhibits a predilection for deleting purine over pyrimidine rNs [77]. This preference mirrors the proofreading control by p53 and could potentially influence the rN mutation spectra induced by HIV-1 RT.

Among all abnormalities in DNA, uracil misincorporation in DNA is an intrinsic factor, resulting in genomic instability, DNA mutations, and apoptosis [78]. During reverse transcription in the cytoplasm, HIV-1 RT inserts non-canonical dUTP with the same efficiency as that of dTTP opposite the template A into the nascent DNA, with subsequent extension of the dU-terminated DNA and the production of a U:A pair (“uracilation”) [69]. Indeed, viral DNA transcripts created in two HIV natural target host cells are heavily uracilated (>500 uracils per 10 kb HIV genome) [79,80]. Remarkably, HIV tolerates, or may perhaps even benefit from, uracil incorporation during the course of reverse transcription in the uracil-rich environment of human immune cells. Uracilation defends HIV DNA from the suicidal pathway of auto-integration, facilitating chromosomal integration and thereby enhancing viral infectivity [80]. Within the context of error-correction events, p53, as a cell model DNA binding protein in the cytoplasm with proofreading utility, may encounter DNA damage and take part in assisting the dU-damage-associated repair path via its skill to exclude preformed 3′-terminal dUs elongated by HIV-1 RT [81]. Upon excision of the non-canonical nucleotide, p53 detaches, thus letting DNA transfer with the correct 3′-terminus to polymerase and the renewal of DNA synthesis. The hallmark of proofreading is the ratio of polymerase to exonuclease activity. The raised dU-editing capacity was perceived through an enhancement in the ratio of p53-exonuclease/ RT-polymerase in the context of activated p53 by drug treatment, e.g., nutlin (a competitive inhibitor of the MDM2-p53 interaction that stimulates p53) [82]. Apparently, the fidelity of DNA synthesis by HIV-1 RT in cytoplasm might react to deviations in the abundance of p53 in the composition of the proviral DNA replication complex [81].

Biochemical data revealed that p53 can donate dual error-correction pathways: avoidance or repair by either decreasing the insertion of non-canonical dUTP into DNA or by promoting the elimination of incorporated dU from nascent DNA [83]. The fact that the functionally specialized exonuclease activity of p53 in mitochondria can extrinsically excise dU incorporated into mitochondrial DNA by pol γ that escapes its own intrinsic exonuclease activity further validates the significant implications of p53 as a prospective proofreader for the removal of dU from HIV DNA in cytoplasm. 

Importantly, HIV-1 RT reveals differences in the extent of incorporation of non-canonical nucleotides during proviral DNA synthesis in two natural target cells [76]. The consequences of a viral infection are always target-cell dependent, and the difference may be explained by inefficient DNA repair response in macrophages compared with lymphocytes. The accuracy of the DNA replication, which comprises the insertion of rN or dU, may be influenced by two opposite and complementary processes: polymerization and proofreading [64,84]. The efficacy of proofreading is an imperious mechanism diminishing the error creation during DNA polymerization, depending upon a number of variables: the physiological fluctuations of the dNTP and rNTP or dUTP pools in the target cell, the ratio of exonuclease/polymerase at the constant dNTP concentrations, and the nature of the lesion [76,79,85]. Excitingly, HIV-1 RT more frequently incorporates non-canonical rNs or dU into the proviral DNA in macrophages, compared with dividing cells, due to the larger cellular concentration disparity between dNTPs and dUTPs or rNTPs [79,86]. Since error correction could result from cellular context-specific effects of the host factors, it is important to comprehend how to link the error-correction functions of p53 in response to HIV infection in two target cells. The potential collaborations between RT enzymology and non-canonical nucleotide repair strategies are highly plausible. It is certainly conceivable that in lymphocytes the low incidence of non-canonical rNs or dU in proviral DNA stems from the defense mechanism executed by p53. Apparently, the virus decision to remove the DNA damage is based on a cell-type-dependent threshold of p53 levels. HIV-triggered considerable upregulation in the amount of p53 in lymphocytes, accompanied by substantial enrichment in the level of exo-competent p53 in cytoplasm, and can result in the low prevalence of non-canonical nucleotides in proviral DNA. Even a low level of error-correction activities contributed by p53 may be valuable for the virus, as the decline in the error rate below a critical threshold may be principally imperative for HIV to escape lethal mutagenesis [81]. Conceivably, this mechanism operates in cytoplasm to avoid the over-uracilation of proviral DNA, thereby positively affecting HIV replication in lymphocytes. 

Notably, there is no elevation in the p53 levels in macrophages upon HIV infection [85]. Accordingly, it is highly expected that the exclusion of non-canonical nucleotides in the cytoplasm by basal p53 might be restrictive due to lower p53-mediated specific error-correction functions in macrophages relative to lymphocytes. In addition, the lower level of human uracil-DNA glycosylases (hUNG) activity in the nucleus of macrophages than in lymphocytes further contributes to substantial enhancement in error rates, and the production of exceptionally mutagenic viruses [81]. In all, the excision of incorporated non-canonical nucleotides by p53 may affect the cell-type-specific infectivity of HIV. Some viruses could remain transcriptionally silenced for years and then become triggered when appropriate stimulatory circumstances are present. Further studies are required to illuminate the distinct functions of p53, specifically in error-correction activities, viral replication, and persistence in host cells. 

### 4.3. Removal of Nucleoside Analogs from DNA by p53 Protein

Antiviral drug development is motivated by the viral life cycle, with antiviral compounds that directly inhibit either viral replication or a cellular process essential for viral replication without affecting critical processes of the host cell. Highly active antiretroviral therapy (HAART) comprises a combination of two nucleoside RT inhibitors (NRTIs), one non-nucleoside RT inhibitor (NNRTI), one integrase strand transfer inhibitor (INSTI), and one protease (PI) inhibitor, each one having different mechanisms of action and interfering with various unique steps of the HIV life cycle in different cell compartments [87]. NRTIs and NNRTIs target different binding pockets near the catalytic site of HIV-1 RT to block the proviral DNA synthesis. NNRTIs can inhibit the RT initiation complex, even during early viral transcription. Integrase inhibitors such as dolutegravir (DTG) and elvitegravir (EVG) target the catalytic site of HIV-1 IN to inhibit the integration of proviral DNA into host genomes [88]. Protease inhibitors such as darunavir (DRV) compete with the natural substrates of HIV-1 protease to inhibit the protease-mediated cleavage of gag and gag-pol precursors. All these treatments can be modified based on induced drug resistance and toxicity [88]. The HIV-1 RT is the primary target for the development of anti-viral drugs, and anti-HIV RT inhibitors are the important components of therapy in clinical practice [87]. Nucleoside analogs (NAs), functioning as prodrugs and after metabolizing becoming pharmacologically active drugs, are the cornerstone of direct-acting anti-viral compounds exploited to treat infection by viruses [89]. NRTIs block chain elongation of the nascent strand by acting as false substrates in the counterfeit incorporation mechanism of HIV-1 RT and cause the stalling of replication forks with a higher possibility of the enzyme dissociation from the template-primer [90]. Clearly, there is a possible linkage between the capacity of an enzyme to insert and extend a wrong nucleotide (resulting in mutagenesis) and to incorporate an NA instead of the correct dNTP (leading to chain termination). Uncovering the mechanisms responsible for DNA repair of NA-induced DNA lesions will have therapeutic value.

The p53 protein was found to bind and edit the nucleoside analogs (NAs) from DNA in vitro, excising the drug molecules from cellular DNA in whole cells with wild-type p53 cells, but not with mutant p53-harboring cells [91]. Excision of therapeutic analogs from cellular DNA by p53 via an intrinsic 3′→5′ exonucleases is presumably a mechanism of drug resistance [91]. The p53 protein in the cytoplasmic compartment can bind 3′ analog-containing DNA and exclude various inserted NAs during the reverse transcription step, albeit less efficiently than the mismatched nucleotides, since longer incubation times were mandatory for the elimination of the terminally incorporated analogs [92]. Despite the success of many approved HIV-1 RT inhibitors, most anti-viral drugs targeted at viral proteins inevitably and frequently develop resistance due to the production of new virus strains produced through acquired mutations in the viral genome [93,94]. Acquired drug resistance mutations can limit anti-retroviral therapy effectiveness. NRTI drug resistance operates in two diverse biochemical routes. The first mechanism relays to the reduced NRTI binding capacity of mutant RTs. The second mechanism relates to the increased exclusion of the chain-terminating NRTI from the 3′ end of the newly synthesized DNA strand, thereby permitting further DNA elongation [94]. These different resistance phenotypes seem to correlate with various sets of mutations in RT. Plausibly, p53 in the cytoplasm, acting as an extrinsic proofreader for NA incorporation, may confer a cellular resistance mechanism to the anti-HIV compounds [91,92].

Of note, during the treatment by NRTIs, in addition to the inhibition of proviral DNA production in cytoplasm, mtDNA synthesis by pol γ in mitochondria is impaired, leading to mitochondrial dysfunction [95]. DNA pol γ, which is responsible for mtDNA replication, repair, and the creation of important enzymes that regulate mitochondrial oxidative phosphorylation and ATP production, has a much higher affinity for NRTIs in comparison to HIV-1 RT and other cellular DNA polymerases at therapeutic doses and is thus more sensitive after chronic/acute exposure to these agents [96]. As a counterpoint to effectiveness, the toxicities of NA are attributable to the failure of the intrinsic proofreading activity of pol γ to proficiently eliminate incorporated chain terminators [97]. The acquired mitochondrial toxicity of most NAs will depend on the incorporation kinetics versus the relative excision rates by the pol γ-associated inherent exonuclease activity [95]. Accumulating evidence indicates that p53 operates as a “guardian of the genome” for mitochondrial DNA synthesis as well [67,98,99]. The fact that p53 may improve the accuracy of DNA synthesis by the excision of NA (thus decreasing their potential for chain termination), in addition to the removal of mispaired nucleotides (thus preventing the production of polymerization errors), implies that this cellular error-correction pathway may compensate for a lack of effective proofreading for pol γ-induced replication errors [66,99]. p53 in the mitochondria holds the potential to be important in defining the cytotoxicity of NAs toward mitochondrial replication, thus affecting the risk–benefit approach (NA toxicity versus viral inhibition) [99].

The increased selectivity for dNTP, or a general reduction in dNTP affinity, leads to a higher replication fidelity. Much higher fidelity-expressing NRTI-resistant mutants, e.g., K65R and M184V, have been described, and their mechanisms of resistance explored [100,101]. The consequences of increased fidelity in vitro include (a) a decrease in the amount of NRTI incorporated during polymerization, (b) a decline in incorrect natural nucleotide incorporation, and (c) a reduction in the efficiency of reverse transcription. Remarkably, altering the fidelity of the mutant RTs, by either increasing or decreasing nucleotide selectivity, has been shown to be detrimental to viral fitness [102]. The rapid production of HIV mutant variants signifies a fitness benefit during the infection of biologically different target cells. The increase in fidelity always comes with a fitness cost, since higher fidelity mutants are replicated more slowly in cell lines. In vivo studies have shown that the M184V/I, K65R, and E89G HIV-1 RT mutants were less fit than parental viruses and often reverted to “wild-type” in the absence of drug treatment [12,100]. Conversely, the effect of lowering fidelity further is predicted to make the genome unstable, producing too many non-viable HIV genomes [103]. It is likely that too high fidelity of DNA synthesis, as well as too low fidelity, negatively affects fitness. The manipulation of viral DNA synthesis accuracy by cellular p53 protein is therefore a possible path for controlling the virus. Understanding the relationship between fidelity and viral fitness will lead to a better appreciation of HIV evolution.

## 5. Concluding Remarks

Genetic diversity and phenotypic variations are intrinsic and peculiar properties of HIV populations. The error rates displayed during copying viral genetic material to produce viral RNA progeny is a biologically relevant parameter of the replication complexes of viruses, exploiting high mutation rates for adaptation to changing environments and expansion of drug resistance [20]. Four parameters that characterize viruses during infection processes can impact disease progression: viral genome replication rate, viral load, genetic heterogeneity, and replicative fitness [103]. Any of them can be targeted for disease control. The consequences of a viral infection are always target-host-dependent.

**Figure 3:**
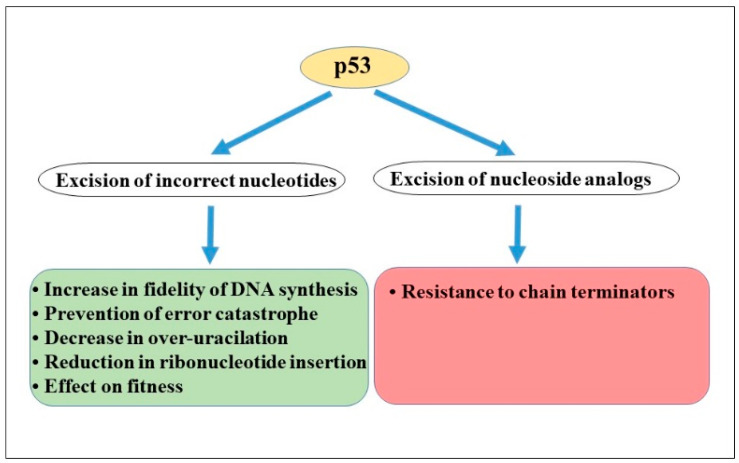
The possible biological outcomes of p53-mediated error correction activities in HIV-infected cells and the consequences of the excision of wrong nucleotides or nucleoside analogs from DNA by p53 protein. See the manuscript text for details.

HIV-infection seeks to take advantage of cellular p53-mediated pathways to generate an adaptive landscape more favorable for virus replication and spreading the virus to neighboring cells. The p53 protein influences a plethora of signaling pathways that regulate various stages of the HIV life cycle by controlling the target cell environment, thus creating an important virus–host interplay that is indispensable for the execution of successful infection [103]. Heterogeneous p53 responses in a target cell to a given HIV infection-mediated stress are accomplished according to the pattern of p53 expression, the precise compartmentalization of the endogenous protein, and its changes in cellular stress situations and timing of the post-translational modifications (e.g., phosphorylation, acetylation), thus eliciting selective programs of either repair of various types of DNA lesions produced during proviral DNA synthesis in the cytoplasm or triggering cell death in the nucleus. p53 elicits diverse cell and/or virus fates during viral infection through two potential models: the subcellular p53 level threshold model and the canonical transcriptional activation model [43]. Apparently, distinct pathways presented by p53 are shaped by context-dependent functionally specialized p53 thresholds during various HIV infection steps: p53 might have a transient interaction with the proviral DNA synthesizing complex in the cytoplasm with subsequent enrichment for the expression of “non-canonical” error correction features, while in the nucleus, by “canonical” function, p53 participates in integration-mediated cell death through transcription-dependent pathways [104]. Within the infected cells, the presence of activated and functional host-coded p53 proteins with concomitant proofreading–repair activities in the cytoplasm may lead to various biological outcomes (Figure 3):

(i) p53 in cytoplasm may serve as a driver proofreader increasing the fidelity of DNA synthesis by HIV-1 RT, thus contributing to the preservation of viral genomic integrity during the incorporation of non-complementary nucleotides [46]. A *bona fide* limitation in the error rates by the p53 proofreader may decrease the production of the unstable viruses in the target cell. 

(ii) Viruses exploit high error rates to adapt to changing environments [103]. Error-prone viral replication places HIV near the threshold of “error catastrophe”. It is highly expected for the virus that, in the presence of cellular fidelity factor exonuclease-competent p53 and subsequent increases in the fidelity of DNA synthesis, the virus could not exceed the tolerable mutation load for viability of a viral population within a host.

(iii) The decrease in the over-uracilation of proviral DNA by the efficient removal of uracils by p53 will determine the proportion of reverse transcripts that undergo suicidal auto-integration in the cytoplasm, thereby affecting viral infectivity [81]. 

(iv) p53, as a stress responder, in the cytoplasm, is capable of facilitating a reduction in the error rates by correcting DNA lesions, including non-canonical ribonucleotides [77].

(v) The degree of HIV RT fidelity is likely to be a balance in which too high fidelity leads to a reduction in viral fitness, but too low fidelity is also detrimental to the propagation of the virus [103]. Hence, manipulation of viral fidelity is therefore a potential path for controlling the virus. Higher fidelity contributed by p53 proofreading activity impacts viral fitness.

(vi) p53 can contribute to the removal of incorporated NA by p53-exonuclease activity, thus conferring decreased sensitivity to anti-viral treatments [83].

The diversity of outcomes that p53 may lead in target cells during host-HIV interaction attests to its significance during viral infection. It is important to further elucidate how other cellular or viral factors/ proteins support or impede p53-proofreader activities during proviral DNA synthesis and other diverse p53-dependent functions.

**Figure 4:**
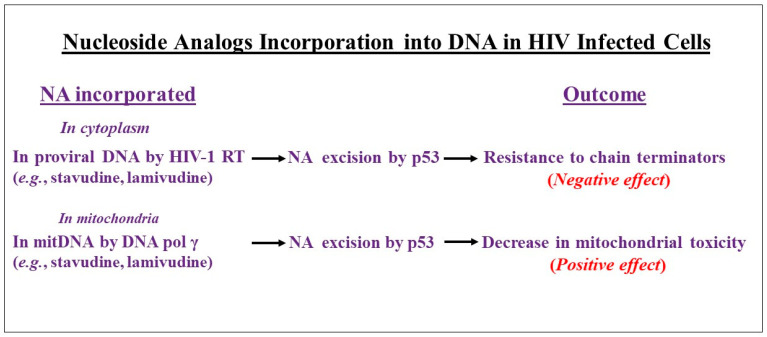
The potential consequences of the removal of incorporated nucleoside analogs (NAs) into DNA in HIV-infected cells by the p53 protein. The excision of NAs by p53 from proviral DNA incorporated by HIV-1 RT in cytoplasm leads to resistance to drugs (negative effect). In mitochondria, the removal of NAs by p53 from mitochondrial DNA (mitDNA) incorporated by DNA pol γ may reduce the potential of chain termination and mitochondrial toxicity (positive effect).

The virus-induced activation of p53 is intricately linked to anti-viral therapy in various compartments of the cell. Increased elimination of chain terminator-NA from drug-containing proviral DNAs in cytoplasm may limit the efficacy of these compounds and facilitate the emergence of drug resistance to anti-viral treatments, which is a negative consequence [92]. However, in mitochondria the excision of NAs from mitDNA by p53 may reduce the potential for chain termination and host toxicity, which is a favorite event for mitochondrial function-positive effects [99] (Figure 4).

A better understandings of the sub-cellular localization of p53 and its role in the maintenance of genetic material in the cytoplasm and drug resistance may create the basis for new strategies in targeted anti-viral therapy that focus on the sub-cellular context of p53 in cells. Future drug screening and superior design of therapeutic approaches, with better efficacy and safety of treatment with low toxicity, should provide higher specificity for HIV-1 RT and lower toxicity for cells (e.g., decrease in drug incorporation by pol γ in mitochondria). Novel therapeutic strategies focusing on the inhibition of the drug-excision reaction would be helpful to combat resistance to multiple nucleoside analogs. Viruses utilize various mechanisms to evade p53-mediated host defenses. p53 function may be regulated by controlling where the protein is in the cell. The dependence on host p53 protein opens up innovative therapeutic avenues, namely, exciting possibilities for developing p53 activators/inhibitors as a next-generation anti-HIV therapy. 

## Data Availability

Data are contained within the article.
